# Crystal structures of tetra­kis­(pyridine-4-thio­amide-κ*N*)bis­(thio­cyanato-κ*N*)cobalt(II) monohydrate and bis­(pyridine-4-thio­amide-κ*N*)bis­(thio­cyanato-κ*N*)zinc(II)

**DOI:** 10.1107/S205698901800021X

**Published:** 2018-01-12

**Authors:** Tristan Neumann, Inke Jess, Christian Näther

**Affiliations:** a Institut für Anorganische Chemie, Christian-Albrechts-Universität Kiel, Max-Eyth Str. 2, D-24118 Kiel, Germany

**Keywords:** crystal structure, discrete complex, cobalt(II) thio­cyanate complex, zinc(II) thio­cyanate complex,4-pyridine­thio­amide, hydrogen bonding

## Abstract

Reaction of Co(NCS)_2_ and Zn(NCS)_2_ with 4-pyridine­thio­amide led to the formation of compounds with composition [Co(NCS)_2_(C_6_H_6_N_2_S)_4_]·H_2_O (**1**) and [Zn(NCS)_2_(C_6_H_6_N_2_S)_2_] (**2**), respectively. The asymmetric unit of compound **1**, consists of one cobalt(II) cation, two thio­cyanate anions, four 4-pyridine­thio­amide ligands and one water mol­ecule whereas that of compound **2** comprises one zinc(II) cation that is located on a twofold rotation axis as well as one thio­cyanate anion and one 4-pyridine­thio­amide ligand in general positions. In the structure of compound **1**, the cobalt(II) cations are octa­hedrally coordinated by two terminal N-bonding thio­cyanate anions and by the N atoms of four 4-pyridine­thio­amide ligands, resulting in discrete and slightly distorted octa­hedral complexes. These complexes are linked into a three-dimensional network *via* inter­molecular N—H⋯S hydrogen bonding between the amino H atoms and the thio­cyanate S atoms. From this arrangement, channels are formed in which the water mol­ecules are embedded and linked to the host structure by inter­molecular O—H⋯S and N—H⋯O hydrogen bonding. In the structure of compound **2**, the zinc(II) cations are tetra­hedrally coordinated by two N-bonding thio­cyanate anions and the N atoms of two 4-pyridine­thio­amide ligands into discrete complexes. These complexes are likewise connected into a three-dimensional network by inter­molecular N—H⋯S hydrogen bonding between the amino H atoms and the thio­amide S atoms.

## Abstract 2

The crystal structures of the title compounds consists of discrete octa­hedral (Co) or tetra­hedral (Zn) complexes that are linked by inter­molecular hydrogen-bonding inter­actions into three-dimensional networks.

## Chemical context   

**Figure 1:**
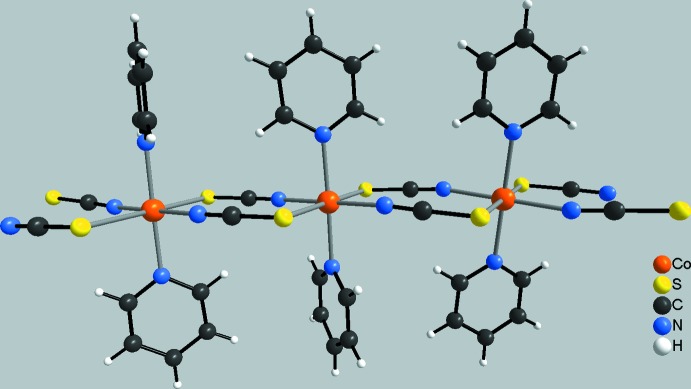
View of a part of a chain in [Co(NCS)_2_(pyridine)_2_]_*n*_ as a representative of compounds with the general composition [*M*(NCS)_2_(*L*)_2_]_*n*_ (*M* = Mn^II^, Fe^II^, Co^II^ or Ni^II^ and *L* = neutral N-donor co-ligand).

Thio- and seleno­cyanate anions are useful ligands for the synthesis of new coordination compounds and polymers, because of their versatile coordination behaviour (Massoud *et al.*, 2013 ▸; Mousavi *et al.*, 2012 ▸; Prananto *et al.*, 2017 ▸; Kabešová *et al.*, 1995 ▸). In this regard, compounds with general composition [*M*(NCS)_2_(*L*)_2_]_*n*_ (*M* = Mn^II^, Fe^II^, Co^II^ or Ni^II^; *L* = neutral N-donor co-ligand) in which the metal cations are linked by these anionic ligands are of special inter­est, because magnetic exchange can be mediated (Palion-Gazda *et al.*, 2015 ▸; Wöhlert *et al.*, 2013*a* ▸). In this context, we are especially inter­ested in cobalt(II) compounds in which the metal cations are octa­hedrally coordinated by two neutral co-ligands and four anionic ligands, which link the central metal cations into chains by pairs of anionic ligands, as symbolized in Fig. 1 ▸. Some of these compounds show a slow relaxation of the magnetization, which in most cases can be traced back to single-chain magnetism (Rams *et al.*, 2017**a* ▸,*b* ▸;* Wöhlert *et al.*, 2012 ▸, 2013*b* ▸). To study the influence of the neutral co-ligand on the magnetic properties, different pyridine derivatives substituted in the 4-position such as 4-benzoyl­pyridine, 4-vinyl­pyridine, 4-acetyl­pyridine, 4-ethyl­pyridine were investigated (Rams *et al.*, 2017*b* ▸; Werner *et al.*, 2015 ▸; Wöhlert *et al.*, 2014 ▸). It was found that all these compounds can be divided magnetically into two groups, even if the same Co(NCS)_2_ chains are observed. In one group, the compounds exhibit an anti­ferromagnetic ground state and the relaxations observed in the magnetic measurements can be attributed to those of single chains. In the second group, the compounds show a ferromagnetic ground state and the relaxations observed at zero field do not correspond to single-chain relaxations. To gain a better insight into this behaviour, additional examinations of such chain compounds are required, which is of extraordinary importance for our project.

Therefore we became inter­ested in the monodentate ligand 4-pyridine­thio­amide. In contrast to all ligands used previously, this ligand might be able to link the Co(NCS)_2_ chains into layers by pairs of inter­molecular hydrogen bonds between the amino H atoms and the thio­amide S atom, which is observed, for example, in the crystal structure of the pure ligand (Colleter & Gadret, 1967 ▸; Eccles *et al.*, 2014 ▸). It should be noted that only one such coordination polymer, namely with 4-pyridine­thio­amide and Cd, is reported in the literature (Neumann *et al.*, 2016 ▸). Here the Cd^II^ cations are linked by pairs of anionic ligands into a linear chain, which corresponds exactly to the structure we are inter­ested in. However, irrespective of the ratio between Co(NCS)_2_ and the co-ligand, a compound with composition Co(NCS)_2_(4-pyridine­thio­amide)_2_ could not be obtained from solution. IR spectroscopic studies of all products showed bands for the CN stretching vibrations at about 2060 cm^−1^, thus indicating only terminal N-coordinating anionic ligands. Therefore the formation of compounds with bridging anionic ligands can be excluded (Bailey *et al.*, 1971 ▸), presumably because cobalt shows no high affinity to bond with sulfur atoms. Hence the formation of discrete complexes with only terminal N-bonding thio­cyanate anions is preferred. The situation is reversed for cadmium, which shows a high affinity to sulfur, and this is obviously the reason why a cadmium compound with a chain structure can easily be obtained from solution. In an alternative approach we tried to synthesize discrete complexes with terminal N-bonding thio­cyanate anions and with additional N-donor co-ligand in the coordination sphere, or mixed ligand complexes with 4-pyridine­thio­amide and other volatile ligands *e.g.* water. Such compounds can easily be transformed into compounds with anion bridges by thermal annealing, as shown previously (Suckert *et al.*, 2017 ▸). In most of these cases, half of the N-bonding co-ligands are replaced by the sulfur atom of the (then bridging) thio­cyanate anion, thus enabling the coordin­ation number of 6 to be maintained. In the course of these investigations, crystals of [Co(NCS)_2_(C_6_H_6_N_2_S)_4_]·H_2_O (**1**) were obtained from aqueous solution and characterized by single crystal X-ray diffraction, which revealed the formation of a discrete complex. Unfortunately, the powder pattern of all batches revealed multi-phase formation, and in several cases large amounts of the 4-pyridine­thio­amide ligand were present in the products (see Fig. S1 in the supporting information).

Co^II^ sometimes forms discrete complexes with composition Co(NCS)_2_(*L*)_2_ in which the cations are tetra­hedrally coordinated by two terminal N-bonding thio­cyanate anions and the N atoms of two neutral co-ligands. In several cases these complexes are isotypic with the corresponding zinc analogues, which enables a simple method for checking whether a tetra­hedral Co complex might be present in the mixture. Hence we synthesized a compound with composition [Zn(NCS)_2_(C_6_H_6_N_2_S)_4_] (**1**) that shows the expected tetra­hedral coordination of zinc(II). However, the calculated X-ray powder diffraction pattern of **2** does not match with the additional reflections observed in some of the X-ray powder diffraction pattern of products obtained during synthesis of **1**. Because of the unknown phase(s), no further investigations were performed.
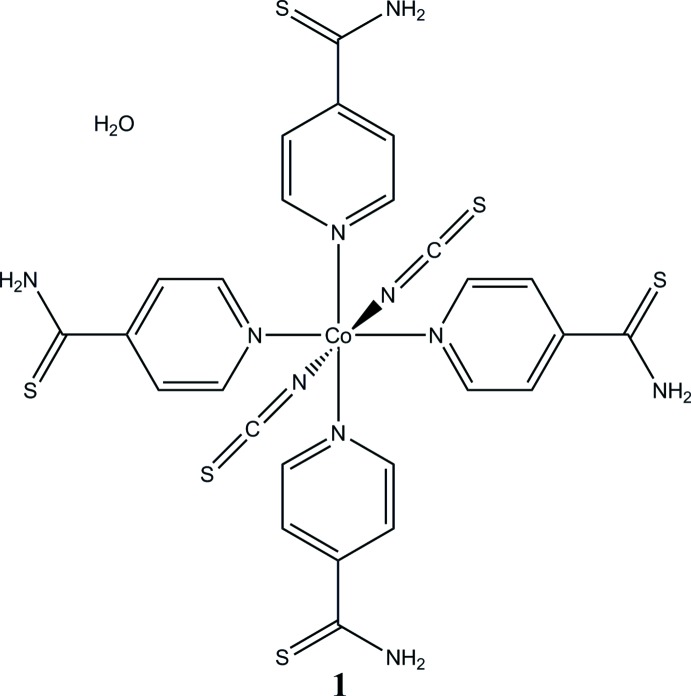
 
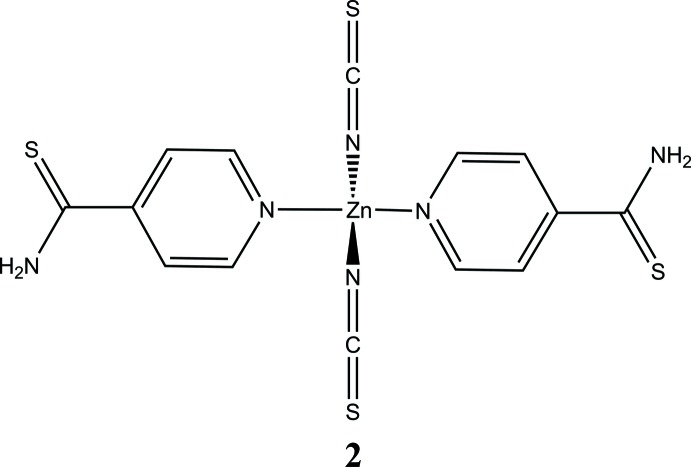


## Structural commentary   

**Figure 2:**
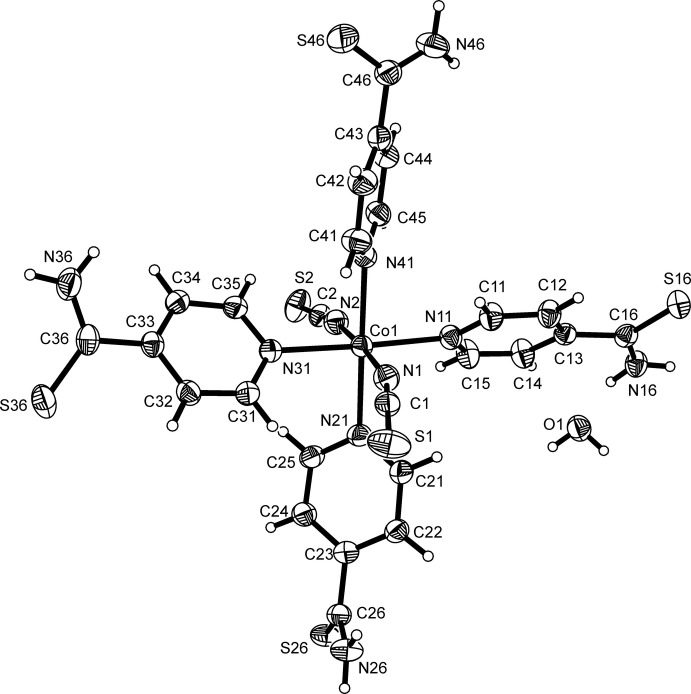
View of the asymmetric unit of compound **1** with the atom labelling and displacement ellipsoids drawn at the 50% probability level.

The asymmetric unit of compound **1** consists of one Co^II^ cation, two thio­cyanate anions, one water mol­ecule and four 4-pyridine­thio­amide co-ligands. The Co^II^ cations are sixfold coordinated by two terminal N-bonding thio­cyanate anions and the N atoms of four 4-pyridine­thio­amide ligands, forming discrete octa­hedral complexes, in which all coordinating atoms are in *trans*-positions (Fig. 2 ▸). This corresponds to the most common arrangement for structures of compounds with general composition *M*(NCS)_2_(*L*)_4_, where *M* is a divalent 3*d* metal cation and *L* a monodentate N-donor co-ligand (Małecki, *et al.*, 2011 ▸). In this context, it is noted that for bridging N-donor co-ligands, like pyrazine or 4,4′-bi­pyridine, two-dimensional networks are obtained, in which the anionic ligands are still terminal coordinating (Real *et al.*, 1991 ▸; Lu *et al.*, 1997 ▸). The Co—N bond lengths to the thio­cyanate anions of 2.0944 (18) and 2.0956 (19) Å are significantly shorter than those to the pyridine N atoms of the 4-pyridine­thio­amide ligand [2.1640 (16) – 2.1761 (16) Å], which is in agreement with related coordination modes reported in the literature (Table 1 ▸; Goodgame *et al.*, 2003 ▸; Prananto *et al.*, 2017 ▸). The bond angles around the central metal cation deviate from the ideal values, indicating a slight distortion (Table 1 ▸). For each co-ligand, the thio­amide group is rotated differently out of the pyridine ring plane, with dihedral angles of 11.8 (2), 55.5 (1), 40.1 (2) and 38.3 (1)°.

**Figure 3:**
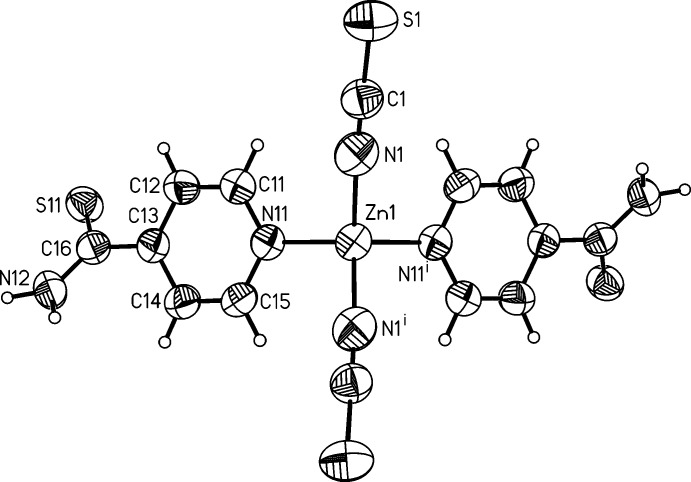
View of the asymmetric unit of compound **2** with the atom labelling and displacement ellipsoids drawn at the 50% probability level. [Symmetry code: (i) *-x* + 3/2, −*y* + 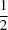, *z*.]

In the structure of compound **2**, the asymmetric unit consists of a Zn^II^ cation that is located on a twofold rotation axis, and one thio­cyanate anion as well as one 4-pyridine­thio­amide ligand in general positions. The Zn^II^ cation is coordinated by the N atoms of two anionic and two neutral co-ligands within a slightly distorted tetra­hedron (Fig. 3 ▸). Bond lengths and angles (Table 2 ▸) are in agreement with values retrieved from the literature. The dihedral angle between the thio­amide group and the pyridine ring is 43.8 (4)°.

## Supra­molecular features   

**Figure 4:**
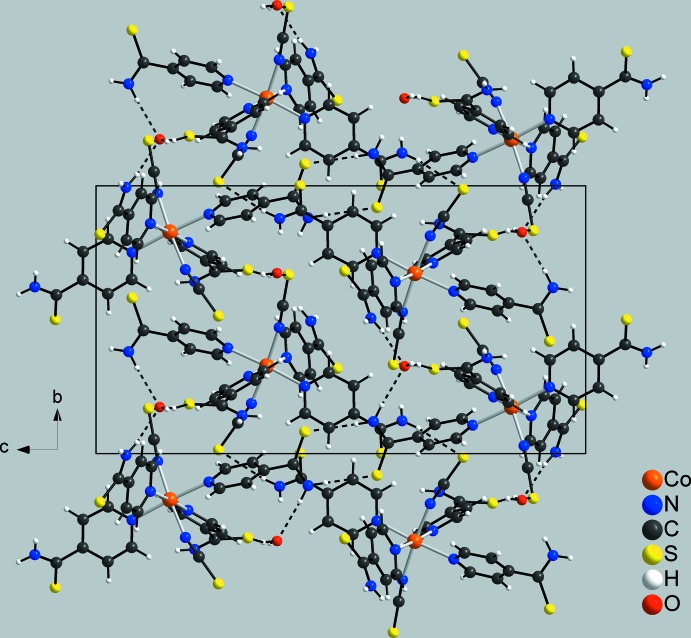
Crystal structure of compound **1** viewed along the *a* axis with inter­molecular hydrogen bonds shown as dashed lines.

In the crystal of compound **1**, the discrete complexes are linked by centrosymmetric pairs of inter­molecular N—H⋯S hydrogen bonds between the amino H atoms and the thio­cyanate S atoms into chains extending parallel to [100], which are further connected by additional N—H⋯S hydrogen bonds into a three-dimensional network (Fig. 4 ▸ and Table 3 ▸). By this arrangement, channels along the *a* axis are formed in which the water mol­ecules are located (Fig. 4 ▸). These solvent mol­ecules are linked to the network *via* inter­molecular O—H⋯S hydrogen bonding between the water H atoms and the thio­cyanate S atoms (Table 3 ▸). The water mol­ecules additionally act as acceptors for N—H⋯O hydrogen bonding to the amino H atoms. There are additional short contacts between some of the aromatic hydrogen atoms and the thio­cyanate S atoms (Table 3 ▸).

**Figure 5:**
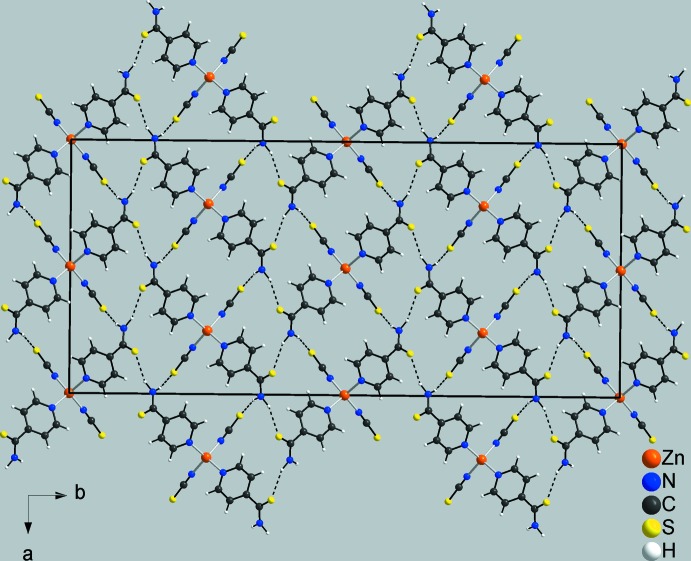
Crystal structure of compound **2** viewed along the *c* axis with inter­molecular hydrogen bonds shown as dashed lines.

In the crystal of compound **2**, the discrete complexes are linked by inter­molecular N—H⋯S hydrogen-bonding interactions between the H atoms of the amino group and thio­amide (S1) and thiocyanate (S11) S atoms, so forming a three-dimensional hydrogen-bonded framework (Fig. 5 ▸ and Table 4 ▸). There is also a weak C15—H15⋯S1^ii^ interaction present within the framework (Table 4 ▸.

## Database survey   

There is only one cobalt thio­cyanate compound with 4-pyridine­thio­amide reported in the Cambridge Structure Database (Version 5.39; Groom *et al.*, 2016 ▸). In tetra­kis(pyri­dine-4-carbo­thio­amide-κ*N* ^1^)bis-(thio­cyanato-κ*N*)cobalt(II) methanol monosolvate, the Co^II^ cations are octa­hedrally coordinated by four pyridine-4-carbo­thio­amide ligands and two thio­cyanate anions, and the solvent mol­ecules are located in the cavities of the structure (Neumann *et al.*, 2017 ▸). Moreover, there is one compound with cadmium, in which the Cd^II^ cations are octa­hedrally coordinated by two terminal N-bonding pyridine­thio­amide ligands and four thio­cyanate anions and linked by pairs of anionic ligands into linear chains (Neumann *et al.*, 2016 ▸). Other coordination compounds with this ligand are unknown. Therefore, the title compound is the third structurally characterized coordination compound with 4-pyridine­thio­amide as a ligand. However, the pure 4-pyridine­thio­amide ligand is also known and in its structure the mol­ecules are linked by pairs of hydrogen bonds between the amino H atoms and the thio­amide S atom (Colleter & Gadret, 1967 ▸; Eccles *et al.*, 2014 ▸). Finally, the protonated form with iodine as counter-anion was reported by Shotonwa & Boeré (2014 ▸).

## Synthesis and crystallization   

Co(NCS)_2_ and 4-pyridine­thio­amide were purchased from Alfa Aesar. Zn(NCS)_2_ was prepared by the reaction of equimolar amounts of Ba(SCN)_2_·3H_2_O with ZnSO_4_·H_2_O in water. The white precipitate of BaSO_4_ was filtered off, and the resulting clear solution was evaporated until complete dryness. The purity of the obtained Zn(NCS)_2_ was checked by X-ray powder diffraction (XRPD) measurements.

Crystals of compound **1** were obtained by the reaction of 8.8 mg of Co(NCS)_2_ (0.05 mmol) with 6.9 mg of 4-pyridine­thio­amide (0.05 mmol) in a mixture of 1 ml of methanol and 1 ml of water. The reaction mixture was heated to boiling and then slowly cooled to ambient temperature, leading to crystals of the title compound suitable for single crystal X-ray diffraction. XRPD revealed impurities by crystals of the employed 4-pyridine­thio­amide ligand as the major phase (see Fig. S1 in the supporting information). Some crystals were selected by hand to measure an infrared spectrum (see Fig. S2 in the supporting information). We also tried to obtain pure samples by using different amounts of Co(NCS)_2_ and 4-pyridine­thio­amide, however without any success.

For the synthesis of compound **2**, 18.2 mg Zn(NCS)_2_ (0.1 mmol) were reacted with 6.9 mg of 4-pyridine­thio­amide (0.05 mmol) in 1.0 ml of water which was then overlayed with 1.0 ml of chloro­form. After a few days, crystals suitable for single crystal X-ray diffraction formed at the inter­face of the solvents.

## Refinement   

Crystal data, data collection and structure refinement details are summarized in Table 5 ▸. For both compounds, the aromatic hydrogen atoms were positioned with idealized geometry and were refined with *U*_iso_(H) = 1.2*U*_eq_(C) using a riding model. The N—H and O—H hydrogen atoms were located in difference-Fourier maps. For compound **1**, their bond lengths were set to ideal values (N—H = 0.88 Å, O—H = 0.84 Å), and refined with *U*_iso_(H) = 1.5*U*_eq_(N,O) using a riding model. For compound **2**, the N—H atoms were initially refined and then held fixed (N—H = 1.01 and 1.03 Å) and refined with *U*_iso_(H) = 1.5*U*_eq_(N,O) using a riding model. The absolute structure of compound **2** was determined by resonant scattering [Flack parameter = 0.014 (18); Table 5 ▸].

## Supplementary Material

Crystal structure: contains datablock(s) 1, 2. DOI: 10.1107/S205698901800021X/wm5430sup1.cif

Structure factors: contains datablock(s) 1. DOI: 10.1107/S205698901800021X/wm54301sup2.hkl

Structure factors: contains datablock(s) 2. DOI: 10.1107/S205698901800021X/wm54302sup3.hkl

Fig. S1 Experimental XRPD pattern of a batch of compound 1 and calculated pattern for 1 and for the 4-pyridinethioamide ligand.. DOI: 10.1107/S205698901800021X/wm5430sup4.pdf

Fig. S2 Infrared spectra of 1, measured from crystals selected by hand. Given is the value of the CN stretching vibration.. DOI: 10.1107/S205698901800021X/wm5430sup5.pdf

CCDC references: 1814632, 1814631

Additional supporting information:  crystallographic information; 3D view; checkCIF report

## Figures and Tables

**Table 1:** Selected geometric parameters (Å, °) for **1** [Chem scheme1]

Co1—N1	2.0944 (18)	Co1—N41	2.1723 (16)
Co1—N2	2.0956 (19)	Co1—N21	2.1730 (16)
Co1—N11	2.1640 (16)	Co1—N31	2.1761 (16)
			
N1—Co1—N2	175.82 (7)	N11—Co1—N21	92.44 (6)
N1—Co1—N11	90.78 (7)	N41—Co1—N21	176.64 (7)
N2—Co1—N11	91.08 (7)	N1—Co1—N31	88.11 (7)
N1—Co1—N41	90.49 (7)	N2—Co1—N31	90.22 (7)
N2—Co1—N41	93.31 (7)	N11—Co1—N31	176.76 (6)
N11—Co1—N41	88.03 (6)	N41—Co1—N31	88.93 (6)
N1—Co1—N21	86.18 (7)	N21—Co1—N31	90.53 (6)
N2—Co1—N21	90.00 (7)		

**Table 2:** Selected geometric parameters (Å, °) for **2** [Chem scheme1]

Zn1—N1^i^	1.935 (6)	Zn1—N11^i^	2.022 (5)
Zn1—N1	1.935 (6)	Zn1—N11	2.023 (5)
			
N1^i^—Zn1—N1	118.4 (4)	N1^i^—Zn1—N11	105.9 (2)
N1^i^—Zn1—N11^i^	106.8 (2)	N1—Zn1—N11	106.8 (2)
N1—Zn1—N11^i^	105.9 (2)	N11^i^—Zn1—N11	113.3 (3)

Symmetry code: (i) 
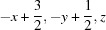
.

**Table 3:** Hydrogen-bond geometry (Å, °) for **1** [Chem scheme1]

*D*—H⋯*A*	*D*—H	H⋯*A*	*D*⋯*A*	*D*—H⋯*A*
C11—H11⋯S46^i^	0.95	2.85	3.674 (2)	145
C12—H12⋯S26^ii^	0.95	2.94	3.695 (2)	137
C14—H14⋯O1	0.95	2.65	3.531 (3)	154
C15—H15⋯S36^iii^	0.95	2.91	3.581 (2)	129
N16—H2*N*⋯O1	0.88	2.06	2.893 (2)	159
C22—H22⋯S36^iv^	0.95	2.96	3.668 (2)	133
N26—H3*N*⋯S26^v^	0.88	2.64	3.5155 (18)	179
N26—H4*N*⋯O1^ii^	0.88	2.21	3.078 (2)	170
N36—H5*N*⋯S26^vi^	0.88	2.78	3.618 (2)	159
N36—H6*N*⋯S1^vii^	0.88	2.96	3.812 (2)	165
C41—H41⋯N1	0.95	2.58	3.104 (3)	115
N46—H7*N*⋯S2^viii^	0.88	2.91	3.782 (2)	174
N46—H8*N*⋯S1^i^	0.88	2.60	3.466 (2)	170
O1—H2*O*1⋯S36^iii^	0.84	2.53	3.2356 (16)	142
O1—H1*O*1⋯S2^iv^	0.84	2.59	3.2394 (16)	135

Symmetry codes: (i) 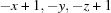; (ii) 
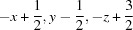
; (iii) 
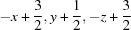
; (iv) 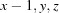; (v) 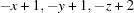; (vi) 
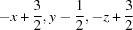
; (vii) 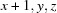; (viii) 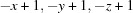.

**Table 4:** Hydrogen-bond geometry (Å, °) for **2** [Chem scheme1]

*D*—H⋯*A*	*D*—H	H⋯*A*	*D*⋯*A*	*D*—H⋯*A*
C15—H15⋯S1^ii^	0.95	2.96	3.690 (6)	135
N12—H1*N*⋯S11^iii^	1.01	2.41	3.358 (5)	156
N12—H2*N*⋯S1^iv^	1.03	2.41	3.424 (6)	166

Symmetry codes: (ii) 
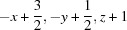
; (iii) 
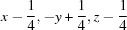
; (iv) 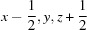.

**Table 5:** Experimental details

	**1**	**2**
Crystal data		
Chemical formula	[Co(NCS)_2_(C_6_H_6_N_2_S)_4_]·H_2_O	[Zn(NCS)_2_(C_6_H_6_N_2_S)_2_]
*M*_r_	745.89	457.91
Crystal system, space group	Monoclinic, *P*2_1_/*n*	Orthorhombic, *F* *d* *d*2
Temperature (K)	200	200
*a*, *b*, *c* (Å)	10.9256 (2), 12.9595 (6), 24.1116 (6)	18.965 (3), 41.216 (7), 5.1117 (7)
α, β, γ (°)	90, 100.763 (2), 90	90, 90, 90
*V* (Å^3^)	3353.91 (19)	3995.6 (11)
*Z*	4	8
Radiation type	Mo *K*α	Mo *K*α
μ (mm^−1^)	0.92	1.66
Crystal size (mm)	0.18 × 0.14 × 0.11	0.11 × 0.08 × 0.06

Data collection		
Diffractometer	Stoe IPDS2	Stoe IPDS2
Absorption correction	Numerical (*X-RED32* and *X-SHAPE*; Stoe, 2008 ▸)	Numerical (*X-RED32* and *X-SHAPE*; Stoe, 2008 ▸)
*T*_min_, *T*_max_	0.787, 0.886	0.789, 0.894
No. of measured, independent and observed [*I* > 2σ(*I*)] reflections	35796, 7301, 6291	6296, 1919, 1711
*R*_int_	0.031	0.087
(sin θ/λ)_max_ (Å^−1^)	0.639	0.617

Refinement		
*R*[*F* ^2^ > 2σ(*F* ^2^)], *wR*(*F* ^2^), *S*	0.036, 0.078, 1.07	0.040, 0.109, 1.07
No. of reflections	7301	1919
No. of parameters	397	114
No. of restraints	0	1
H-atom treatment	H-atom parameters constrained	H-atom parameters constrained
Δρ_max_, Δρ_min_ (e Å^−3^)	0.37, −0.38	0.41, −0.36
Absolute structure	–	Flack *x* determined using 638 quotients [(*I* ^+^)−(*I* ^−^)]/[(*I* ^+^)+(*I* ^−^)] (Parsons *et al.*, 2013 ▸)
Absolute structure parameter	–	0.014 (18)

Computer programs: *X-AREA* (Stoe, 2008 ▸), *SHELXS97* (Sheldrick, 2008 ▸), *SHELXL2014* (Sheldrick, 2015 ▸), *DIAMOND* (Brandenburg, 1990 ▸) and *publCIF* (Westrip, 2010 ▸).

## supplementary crystallographic information

### Tetrakis(pyridine-4-thioamide-κN)bis(thiocyanato-κN)cobalt(II) monohydrate (1) Crystal data

**Table:**

[Co(NCS)_2_(C_6_H_6_N_2_S)_4_]·H_2_O	*F*(000) = 1532
*M**_r_* = 745.89	*D*_x_ = 1.477 Mg m^−^^3^
Monoclinic, *P*2_1_/*n*	Mo *K*α radiation, λ = 0.71073 Å
*a* = 10.9256 (2) Å	Cell parameters from 7301 reflections
*b* = 12.9595 (6) Å	θ = 3.1–54.0°
*c* = 24.1116 (6) Å	µ = 0.92 mm^−^^1^
β = 100.763 (2)°	*T* = 200 K
*V* = 3353.91 (19) Å^3^	Block, light red
*Z* = 4	0.18 × 0.14 × 0.11 mm

### Tetrakis(pyridine-4-thioamide-κN)bis(thiocyanato-κN)cobalt(II) monohydrate (1) Data collection

**Table:**

Stoe IPDS-2 diffractometer	6291 reflections with *I* > 2σ(*I*)
ω scans	*R*_int_ = 0.031
Absorption correction: numerical (*X-RED32* and *X-SHAPE*; Stoe, 2008)	θ_max_ = 27.0°, θ_min_ = 1.7°
*T*_min_ = 0.787, *T*_max_ = 0.886	*h* = −13→13
35796 measured reflections	*k* = −16→16
7301 independent reflections	*l* = −30→30

### Tetrakis(pyridine-4-thioamide-κN)bis(thiocyanato-κN)cobalt(II) monohydrate (1) Refinement

**Table:**

Refinement on *F*^2^	0 restraints
Least-squares matrix: full	Hydrogen site location: mixed
*R*[*F*^2^ > 2σ(*F*^2^)] = 0.036	H-atom parameters constrained
*wR*(*F*^2^) = 0.078	*w* = 1/[σ^2^(*F*_o_^2^) + (0.033*P*)^2^ + 1.8302*P*] where *P* = (*F*_o_^2^ + 2*F*_c_^2^)/3
*S* = 1.07	(Δ/σ)_max_ = 0.001
7301 reflections	Δρ_max_ = 0.37 e Å^−^^3^
397 parameters	Δρ_min_ = −0.38 e Å^−^^3^

### Tetrakis(pyridine-4-thioamide-κN)bis(thiocyanato-κN)cobalt(II) monohydrate (1) Special details

**Table:**

Geometry. All esds (except the esd in the dihedral angle between two l.s. planes) are estimated using the full covariance matrix. The cell esds are taken into account individually in the estimation of esds in distances, angles and torsion angles; correlations between esds in cell parameters are only used when they are defined by crystal symmetry. An approximate (isotropic) treatment of cell esds is used for estimating esds involving l.s. planes.

### Tetrakis(pyridine-4-thioamide-κN)bis(thiocyanato-κN)cobalt(II) monohydrate (1) Fractional atomic coordinates and isotropic or equivalent isotropic displacement parameters (Å^2^)

**Table:**

	*x*	*y*	*z*	*U*_iso_*/*U*_eq_	
Co1	0.56386 (2)	0.32716 (2)	0.65116 (2)	0.02828 (7)	
N1	0.50992 (16)	0.18701 (14)	0.68256 (8)	0.0350 (4)	
C1	0.50476 (18)	0.11498 (17)	0.71023 (9)	0.0334 (4)	
S1	0.49655 (7)	0.01363 (5)	0.74981 (3)	0.05452 (17)	
N2	0.62178 (16)	0.47098 (14)	0.62584 (8)	0.0362 (4)	
C2	0.65867 (18)	0.55240 (17)	0.61831 (9)	0.0340 (4)	
S2	0.71112 (5)	0.66765 (5)	0.60707 (3)	0.05095 (16)	
N11	0.37335 (14)	0.35882 (13)	0.61109 (7)	0.0310 (3)	
C11	0.30074 (18)	0.28333 (16)	0.58515 (9)	0.0353 (4)	
H11	0.3365	0.2171	0.5823	0.042*	
C12	0.17590 (18)	0.29742 (16)	0.56226 (9)	0.0356 (4)	
H12	0.1281	0.2420	0.5436	0.043*	
C13	0.12101 (17)	0.39272 (15)	0.56661 (8)	0.0284 (4)	
C14	0.19731 (19)	0.47126 (16)	0.59235 (10)	0.0383 (5)	
H14	0.1644	0.5384	0.5955	0.046*	
C15	0.32153 (19)	0.45127 (17)	0.61330 (10)	0.0388 (5)	
H15	0.3726	0.5064	0.6302	0.047*	
C16	−0.01659 (17)	0.40709 (15)	0.54605 (8)	0.0309 (4)	
S16	−0.10407 (5)	0.31858 (4)	0.50805 (2)	0.03728 (12)	
N16	−0.06492 (15)	0.49408 (14)	0.56133 (8)	0.0361 (4)	
H1N	−0.1449	0.5073	0.5508	0.054*	
H2N	−0.0220	0.5408	0.5833	0.054*	
N21	0.53727 (15)	0.39399 (13)	0.73071 (7)	0.0318 (3)	
C21	0.43666 (18)	0.37052 (17)	0.75246 (9)	0.0348 (4)	
H21	0.3684	0.3381	0.7286	0.042*	
C22	0.42671 (19)	0.39088 (17)	0.80772 (9)	0.0344 (4)	
H22	0.3534	0.3730	0.8214	0.041*	
C23	0.52596 (18)	0.43786 (15)	0.84273 (8)	0.0305 (4)	
C24	0.62852 (19)	0.46660 (17)	0.82001 (9)	0.0354 (4)	
H24	0.6963	0.5020	0.8425	0.042*	
C25	0.63077 (19)	0.44304 (16)	0.76421 (9)	0.0337 (4)	
H25	0.7017	0.4625	0.7491	0.040*	
C26	0.51995 (18)	0.45985 (16)	0.90307 (8)	0.0322 (4)	
S26	0.53825 (5)	0.58106 (4)	0.92626 (2)	0.03722 (12)	
N26	0.49702 (18)	0.38057 (14)	0.93325 (8)	0.0396 (4)	
H3N	0.4892	0.3904	0.9685	0.059*	
H4N	0.4850	0.3200	0.9167	0.059*	
N31	0.75564 (14)	0.28842 (13)	0.68796 (7)	0.0302 (3)	
C31	0.78932 (18)	0.23749 (16)	0.73696 (8)	0.0333 (4)	
H31	0.7276	0.2243	0.7591	0.040*	
C32	0.90894 (18)	0.20355 (16)	0.75667 (9)	0.0328 (4)	
H32	0.9293	0.1692	0.7920	0.039*	
C33	0.99949 (17)	0.22025 (15)	0.72412 (8)	0.0298 (4)	
C34	0.96583 (18)	0.27467 (16)	0.67424 (8)	0.0322 (4)	
H34	1.0257	0.2890	0.6513	0.039*	
C35	0.84442 (18)	0.30784 (16)	0.65808 (8)	0.0323 (4)	
H35	0.8229	0.3463	0.6241	0.039*	
C36	1.12841 (18)	0.17958 (16)	0.74238 (9)	0.0344 (4)	
S36	1.19683 (5)	0.18531 (5)	0.80975 (3)	0.04698 (14)	
N36	1.18076 (17)	0.14209 (16)	0.70151 (9)	0.0458 (5)	
H5N	1.1414	0.1379	0.6662	0.069*	
H6N	1.2580	0.1195	0.7067	0.069*	
N41	0.58869 (15)	0.25150 (13)	0.57362 (7)	0.0324 (4)	
C41	0.6140 (2)	0.15032 (17)	0.57289 (9)	0.0396 (5)	
H41	0.6274	0.1137	0.6076	0.048*	
C42	0.6215 (2)	0.09683 (18)	0.52419 (9)	0.0403 (5)	
H42	0.6413	0.0254	0.5257	0.048*	
C43	0.59981 (17)	0.14867 (17)	0.47296 (9)	0.0337 (4)	
C44	0.57594 (19)	0.25345 (17)	0.47372 (9)	0.0364 (4)	
H44	0.5627	0.2920	0.4396	0.044*	
C45	0.57158 (19)	0.30155 (16)	0.52443 (9)	0.0345 (4)	
H45	0.5556	0.3736	0.5243	0.041*	
C46	0.60338 (19)	0.09230 (17)	0.41907 (9)	0.0374 (5)	
S46	0.70646 (6)	0.00009 (5)	0.41673 (3)	0.05019 (15)	
N46	0.51894 (19)	0.12246 (17)	0.37506 (8)	0.0481 (5)	
H7N	0.4664	0.1733	0.3767	0.072*	
H8N	0.5204	0.0945	0.3419	0.072*	
O1	0.01279 (14)	0.67371 (12)	0.63019 (7)	0.0425 (4)	
H2O1	0.0695	0.6686	0.6588	0.064*	
H1O1	−0.0504	0.6648	0.6448	0.064*	

### Tetrakis(pyridine-4-thioamide-κN)bis(thiocyanato-κN)cobalt(II) monohydrate (1) Atomic displacement parameters (Å^2^)

**Table:**

	*U*^11^	*U*^22^	*U*^33^	*U*^12^	*U*^13^	*U*^23^
Co1	0.02703 (12)	0.03082 (14)	0.02647 (13)	0.00297 (10)	0.00368 (9)	−0.00167 (10)
N1	0.0347 (8)	0.0343 (9)	0.0356 (9)	−0.0011 (7)	0.0053 (7)	−0.0004 (7)
C1	0.0310 (9)	0.0368 (11)	0.0320 (10)	0.0019 (8)	0.0050 (8)	−0.0077 (9)
S1	0.0897 (5)	0.0383 (3)	0.0367 (3)	−0.0010 (3)	0.0148 (3)	0.0037 (2)
N2	0.0357 (9)	0.0364 (10)	0.0364 (9)	0.0014 (7)	0.0068 (7)	0.0007 (7)
C2	0.0276 (9)	0.0423 (12)	0.0330 (10)	0.0064 (8)	0.0076 (8)	0.0012 (9)
S2	0.0403 (3)	0.0413 (3)	0.0735 (4)	0.0019 (2)	0.0164 (3)	0.0135 (3)
N11	0.0283 (8)	0.0305 (8)	0.0332 (9)	0.0038 (6)	0.0033 (6)	−0.0007 (7)
C11	0.0310 (10)	0.0292 (10)	0.0449 (12)	0.0031 (8)	0.0050 (8)	−0.0045 (9)
C12	0.0305 (9)	0.0309 (11)	0.0448 (12)	−0.0018 (8)	0.0056 (8)	−0.0040 (9)
C13	0.0289 (9)	0.0295 (10)	0.0271 (9)	0.0004 (7)	0.0060 (7)	0.0030 (7)
C14	0.0341 (10)	0.0283 (10)	0.0496 (13)	0.0050 (8)	0.0002 (9)	−0.0029 (9)
C15	0.0327 (10)	0.0311 (11)	0.0492 (13)	0.0029 (8)	−0.0010 (9)	−0.0074 (9)
C16	0.0309 (9)	0.0316 (10)	0.0311 (10)	−0.0001 (8)	0.0079 (8)	0.0065 (8)
S16	0.0319 (2)	0.0361 (3)	0.0416 (3)	−0.0025 (2)	0.0012 (2)	−0.0011 (2)
N16	0.0287 (8)	0.0348 (9)	0.0441 (10)	0.0032 (7)	0.0056 (7)	−0.0021 (8)
N21	0.0340 (8)	0.0332 (9)	0.0291 (8)	0.0002 (7)	0.0083 (7)	−0.0033 (7)
C21	0.0311 (9)	0.0407 (11)	0.0331 (10)	−0.0035 (8)	0.0073 (8)	−0.0054 (9)
C22	0.0330 (9)	0.0380 (11)	0.0337 (10)	−0.0050 (8)	0.0104 (8)	−0.0022 (8)
C23	0.0365 (10)	0.0264 (9)	0.0297 (10)	0.0024 (8)	0.0089 (8)	0.0009 (7)
C24	0.0363 (10)	0.0363 (11)	0.0337 (10)	−0.0074 (8)	0.0069 (8)	−0.0058 (8)
C25	0.0352 (10)	0.0350 (11)	0.0333 (10)	−0.0051 (8)	0.0121 (8)	−0.0050 (8)
C26	0.0344 (10)	0.0342 (11)	0.0284 (10)	0.0009 (8)	0.0070 (8)	0.0004 (8)
S26	0.0519 (3)	0.0308 (3)	0.0302 (2)	−0.0042 (2)	0.0112 (2)	−0.0028 (2)
N26	0.0589 (11)	0.0319 (9)	0.0301 (9)	−0.0003 (8)	0.0136 (8)	0.0011 (7)
N31	0.0277 (7)	0.0349 (9)	0.0278 (8)	0.0033 (7)	0.0047 (6)	0.0004 (7)
C31	0.0301 (9)	0.0403 (11)	0.0297 (10)	−0.0003 (8)	0.0060 (7)	0.0031 (8)
C32	0.0316 (9)	0.0362 (11)	0.0292 (10)	0.0002 (8)	0.0022 (8)	0.0037 (8)
C33	0.0277 (9)	0.0268 (9)	0.0338 (10)	−0.0011 (7)	0.0028 (7)	−0.0025 (8)
C34	0.0307 (9)	0.0355 (11)	0.0316 (10)	0.0008 (8)	0.0084 (8)	0.0008 (8)
C35	0.0329 (9)	0.0369 (11)	0.0274 (9)	0.0022 (8)	0.0062 (7)	0.0021 (8)
C36	0.0276 (9)	0.0282 (10)	0.0461 (12)	−0.0017 (8)	0.0032 (8)	0.0020 (9)
S36	0.0340 (3)	0.0548 (4)	0.0469 (3)	0.0003 (2)	−0.0058 (2)	0.0079 (3)
N36	0.0307 (9)	0.0502 (12)	0.0555 (12)	0.0077 (8)	0.0058 (8)	−0.0066 (9)
N41	0.0303 (8)	0.0368 (9)	0.0290 (8)	0.0061 (7)	0.0031 (6)	−0.0039 (7)
C41	0.0467 (12)	0.0394 (12)	0.0323 (11)	0.0134 (9)	0.0062 (9)	0.0002 (9)
C42	0.0440 (11)	0.0372 (12)	0.0398 (12)	0.0080 (9)	0.0079 (9)	−0.0042 (9)
C43	0.0269 (9)	0.0396 (11)	0.0341 (10)	−0.0016 (8)	0.0048 (8)	−0.0067 (8)
C44	0.0373 (10)	0.0389 (11)	0.0325 (10)	−0.0009 (9)	0.0050 (8)	−0.0018 (9)
C45	0.0354 (10)	0.0342 (11)	0.0327 (10)	0.0012 (8)	0.0034 (8)	−0.0025 (8)
C46	0.0373 (10)	0.0399 (12)	0.0367 (11)	−0.0070 (9)	0.0111 (9)	−0.0066 (9)
S46	0.0470 (3)	0.0524 (4)	0.0524 (4)	0.0055 (3)	0.0126 (3)	−0.0179 (3)
N46	0.0593 (12)	0.0506 (12)	0.0334 (10)	0.0037 (10)	0.0060 (9)	−0.0092 (9)
O1	0.0393 (8)	0.0445 (9)	0.0423 (9)	−0.0037 (7)	0.0037 (7)	−0.0009 (7)

### Tetrakis(pyridine-4-thioamide-κN)bis(thiocyanato-κN)cobalt(II) monohydrate (1) Geometric parameters (Å, º)

**Table:**

Co1—N1	2.0944 (18)	C26—N26	1.310 (3)
Co1—N2	2.0956 (19)	C26—S26	1.667 (2)
Co1—N11	2.1640 (16)	N26—H3N	0.8801
Co1—N41	2.1723 (16)	N26—H4N	0.8800
Co1—N21	2.1730 (16)	N31—C35	1.336 (2)
Co1—N31	2.1761 (16)	N31—C31	1.343 (3)
N1—C1	1.155 (3)	C31—C32	1.377 (3)
C1—S1	1.636 (2)	C31—H31	0.9500
N2—C2	1.156 (3)	C32—C33	1.390 (3)
C2—S2	1.640 (2)	C32—H32	0.9500
N11—C15	1.331 (3)	C33—C34	1.384 (3)
N11—C11	1.339 (3)	C33—C36	1.491 (3)
C11—C12	1.385 (3)	C34—C35	1.380 (3)
C11—H11	0.9500	C34—H34	0.9500
C12—C13	1.385 (3)	C35—H35	0.9500
C12—H12	0.9500	C36—N36	1.321 (3)
C13—C14	1.387 (3)	C36—S36	1.658 (2)
C13—C16	1.504 (3)	N36—H5N	0.8803
C14—C15	1.381 (3)	N36—H6N	0.8800
C14—H14	0.9500	N41—C45	1.334 (3)
C15—H15	0.9500	N41—C41	1.341 (3)
C16—N16	1.326 (3)	C41—C42	1.379 (3)
C16—S16	1.656 (2)	C41—H41	0.9500
N16—H1N	0.8799	C42—C43	1.387 (3)
N16—H2N	0.8800	C42—H42	0.9500
N21—C21	1.338 (3)	C43—C44	1.384 (3)
N21—C25	1.339 (3)	C43—C46	1.497 (3)
C21—C22	1.382 (3)	C44—C45	1.381 (3)
C21—H21	0.9500	C44—H44	0.9500
C22—C23	1.385 (3)	C45—H45	0.9500
C22—H22	0.9500	C46—N46	1.328 (3)
C23—C24	1.387 (3)	C46—S46	1.650 (2)
C23—C26	1.496 (3)	N46—H7N	0.8800
C24—C25	1.384 (3)	N46—H8N	0.8802
C24—H24	0.9500	O1—H2O1	0.8400
C25—H25	0.9500	O1—H1O1	0.8400
			
N1—Co1—N2	175.82 (7)	N21—C25—C24	122.76 (18)
N1—Co1—N11	90.78 (7)	N21—C25—H25	118.6
N2—Co1—N11	91.08 (7)	C24—C25—H25	118.6
N1—Co1—N41	90.49 (7)	N26—C26—C23	116.01 (18)
N2—Co1—N41	93.31 (7)	N26—C26—S26	125.19 (16)
N11—Co1—N41	88.03 (6)	C23—C26—S26	118.78 (15)
N1—Co1—N21	86.18 (7)	C26—N26—H3N	119.2
N2—Co1—N21	90.00 (7)	C26—N26—H4N	118.4
N11—Co1—N21	92.44 (6)	H3N—N26—H4N	122.3
N41—Co1—N21	176.64 (7)	C35—N31—C31	117.37 (17)
N1—Co1—N31	88.11 (7)	C35—N31—Co1	118.52 (13)
N2—Co1—N31	90.22 (7)	C31—N31—Co1	123.83 (13)
N11—Co1—N31	176.76 (6)	N31—C31—C32	123.17 (18)
N41—Co1—N31	88.93 (6)	N31—C31—H31	118.4
N21—Co1—N31	90.53 (6)	C32—C31—H31	118.4
C1—N1—Co1	163.19 (17)	C31—C32—C33	119.03 (18)
N1—C1—S1	179.4 (2)	C31—C32—H32	120.5
C2—N2—Co1	172.22 (18)	C33—C32—H32	120.5
N2—C2—S2	179.5 (2)	C34—C33—C32	117.95 (17)
C15—N11—C11	117.21 (17)	C34—C33—C36	121.32 (18)
C15—N11—Co1	122.27 (14)	C32—C33—C36	120.73 (18)
C11—N11—Co1	120.44 (13)	C35—C34—C33	119.33 (18)
N11—C11—C12	122.93 (19)	C35—C34—H34	120.3
N11—C11—H11	118.5	C33—C34—H34	120.3
C12—C11—H11	118.5	N31—C35—C34	123.07 (18)
C11—C12—C13	119.70 (19)	N31—C35—H35	118.5
C11—C12—H12	120.1	C34—C35—H35	118.5
C13—C12—H12	120.1	N36—C36—C33	115.24 (19)
C12—C13—C14	117.11 (18)	N36—C36—S36	124.65 (16)
C12—C13—C16	120.37 (18)	C33—C36—S36	120.10 (16)
C14—C13—C16	122.48 (18)	C36—N36—H5N	122.8
C15—C14—C13	119.58 (19)	C36—N36—H6N	123.6
C15—C14—H14	120.2	H5N—N36—H6N	113.6
C13—C14—H14	120.2	C45—N41—C41	117.31 (18)
N11—C15—C14	123.4 (2)	C45—N41—Co1	121.76 (14)
N11—C15—H15	118.3	C41—N41—Co1	120.79 (14)
C14—C15—H15	118.3	N41—C41—C42	123.2 (2)
N16—C16—C13	116.05 (18)	N41—C41—H41	118.4
N16—C16—S16	121.60 (15)	C42—C41—H41	118.4
C13—C16—S16	122.33 (15)	C41—C42—C43	119.2 (2)
C16—N16—H1N	120.7	C41—C42—H42	120.4
C16—N16—H2N	123.8	C43—C42—H42	120.4
H1N—N16—H2N	115.4	C44—C43—C42	117.70 (19)
C21—N21—C25	117.49 (17)	C44—C43—C46	121.7 (2)
C21—N21—Co1	120.68 (14)	C42—C43—C46	120.6 (2)
C25—N21—Co1	120.61 (13)	C45—C44—C43	119.5 (2)
N21—C21—C22	123.54 (19)	C45—C44—H44	120.2
N21—C21—H21	118.2	C43—C44—H44	120.2
C22—C21—H21	118.2	N41—C45—C44	123.1 (2)
C21—C22—C23	118.55 (18)	N41—C45—H45	118.5
C21—C22—H22	120.7	C44—C45—H45	118.5
C23—C22—H22	120.7	N46—C46—C43	115.02 (19)
C22—C23—C24	118.41 (18)	N46—C46—S46	124.22 (17)
C22—C23—C26	120.34 (17)	C43—C46—S46	120.76 (17)
C24—C23—C26	121.22 (18)	C46—N46—H7N	123.1
C25—C24—C23	119.14 (19)	C46—N46—H8N	118.4
C25—C24—H24	120.4	H7N—N46—H8N	118.1
C23—C24—H24	120.4	H2O1—O1—H1O1	100.5

### Tetrakis(pyridine-4-thioamide-κN)bis(thiocyanato-κN)cobalt(II) monohydrate (1) Hydrogen-bond geometry (Å, º)

**Table:**

*D*—H···*A*	*D*—H	H···*A*	*D*···*A*	*D*—H···*A*
C11—H11···S46^i^	0.95	2.85	3.674 (2)	145
C12—H12···S26^ii^	0.95	2.94	3.695 (2)	137
C14—H14···O1	0.95	2.65	3.531 (3)	154
C15—H15···S36^iii^	0.95	2.91	3.581 (2)	129
N16—H2*N*···O1	0.88	2.06	2.893 (2)	159
C22—H22···S36^iv^	0.95	2.96	3.668 (2)	133
N26—H3*N*···S26^v^	0.88	2.64	3.5155 (18)	179
N26—H4*N*···O1^ii^	0.88	2.21	3.078 (2)	170
N36—H5*N*···S26^vi^	0.88	2.78	3.618 (2)	159
N36—H6*N*···S1^vii^	0.88	2.96	3.812 (2)	165
C41—H41···N1	0.95	2.58	3.104 (3)	115
N46—H7*N*···S2^viii^	0.88	2.91	3.782 (2)	174
N46—H8*N*···S1^i^	0.88	2.60	3.466 (2)	170
O1—H2*O*1···S36^iii^	0.84	2.53	3.2356 (16)	142
O1—H1*O*1···S2^iv^	0.84	2.59	3.2394 (16)	135

Symmetry codes: (i) −*x*+1, −*y*, −*z*+1; (ii) −*x*+1/2, *y*−1/2, −*z*+3/2; (iii) −*x*+3/2, *y*+1/2, −*z*+3/2; (iv) *x*−1, *y*, *z*; (v) −*x*+1, −*y*+1, −*z*+2; (vi) −*x*+3/2, *y*−1/2, −*z*+3/2; (vii) *x*+1, *y*, *z*; (viii) −*x*+1, −*y*+1, −*z*+1.

### Bis(pyridine-4-thioamide-κN)bis(thiocyanato-κN)zinc(II) (2) Crystal data

**Table:**

[Zn(NCS)_2_(C_6_H_6_N_2_S)_2_]	*D*_x_ = 1.522 Mg m^−^^3^
*M**_r_* = 457.91	Mo *K*α radiation, λ = 0.71073 Å
Orthorhombic, *F**d**d*2	Cell parameters from 6296 reflections
*a* = 18.965 (3) Å	θ = 2.0–26.0°
*b* = 41.216 (7) Å	µ = 1.66 mm^−^^1^
*c* = 5.1117 (7) Å	*T* = 200 K
*V* = 3995.6 (11) Å^3^	Block, colorless
*Z* = 8	0.11 × 0.08 × 0.06 mm
*F*(000) = 1856	

### Bis(pyridine-4-thioamide-κN)bis(thiocyanato-κN)zinc(II) (2) Data collection

**Table:**

Stoe IPDS-2 diffractometer	1711 reflections with *I* > 2σ(*I*)
ω scans	*R*_int_ = 0.087
Absorption correction: numerical (*X-RED32* and *X-SHAPE*; Stoe, 2008)	θ_max_ = 26.0°, θ_min_ = 2.0°
*T*_min_ = 0.789, *T*_max_ = 0.894	*h* = −23→23
6296 measured reflections	*k* = −50→50
1919 independent reflections	*l* = −5→6

### Bis(pyridine-4-thioamide-κN)bis(thiocyanato-κN)zinc(II) (2) Refinement

**Table:**

Refinement on *F*^2^	Hydrogen site location: mixed
Least-squares matrix: full	H-atom parameters constrained
*R*[*F*^2^ > 2σ(*F*^2^)] = 0.040	*w* = 1/[σ^2^(*F*_o_^2^) + (0.0506*P*)^2^ + 3.6629*P*] where *P* = (*F*_o_^2^ + 2*F*_c_^2^)/3
*wR*(*F*^2^) = 0.109	(Δ/σ)_max_ < 0.001
*S* = 1.07	Δρ_max_ = 0.41 e Å^−^^3^
1919 reflections	Δρ_min_ = −0.36 e Å^−^^3^
114 parameters	Absolute structure: Flack *x* determined using 638 quotients [(*I*^+^)-(*I*^-^)]/[(*I*^+^)+(*I*^-^)] (Parsons *et al.*, 2013)
1 restraint	Absolute structure parameter: 0.014 (18)

### Bis(pyridine-4-thioamide-κN)bis(thiocyanato-κN)zinc(II) (2) Special details

**Table:**

Geometry. All esds (except the esd in the dihedral angle between two l.s. planes) are estimated using the full covariance matrix. The cell esds are taken into account individually in the estimation of esds in distances, angles and torsion angles; correlations between esds in cell parameters are only used when they are defined by crystal symmetry. An approximate (isotropic) treatment of cell esds is used for estimating esds involving l.s. planes.

### Bis(pyridine-4-thioamide-κN)bis(thiocyanato-κN)zinc(II) (2) Fractional atomic coordinates and isotropic or equivalent isotropic displacement parameters (Å^2^)

**Table:**

	*x*	*y*	*z*	*U*_iso_*/*U*_eq_	
Zn1	0.7500	0.2500	0.4335 (2)	0.0566 (3)	
N1	0.8164 (3)	0.22371 (14)	0.2398 (12)	0.0719 (17)	
C1	0.8585 (3)	0.20935 (15)	0.1215 (13)	0.0598 (15)	
S1	0.91578 (10)	0.18998 (5)	−0.0532 (5)	0.0774 (5)	
N11	0.6912 (2)	0.21920 (10)	0.6510 (12)	0.0554 (12)	
C11	0.7150 (3)	0.18989 (13)	0.7237 (13)	0.0572 (14)	
H11	0.7618	0.1839	0.6781	0.069*	
C12	0.6743 (3)	0.16827 (14)	0.8614 (13)	0.0601 (15)	
H12	0.6932	0.1479	0.9129	0.072*	
C13	0.6053 (3)	0.17635 (12)	0.9247 (14)	0.0550 (12)	
C14	0.5813 (3)	0.20657 (14)	0.8525 (14)	0.0616 (16)	
H14	0.5348	0.2131	0.8962	0.074*	
C15	0.6250 (3)	0.22727 (13)	0.7169 (15)	0.0645 (16)	
H15	0.6076	0.2480	0.6681	0.077*	
C16	0.5580 (3)	0.15290 (13)	1.0657 (13)	0.0563 (14)	
N12	0.4930 (3)	0.15183 (13)	0.9681 (12)	0.0618 (13)	
H1N	0.4537	0.1374	1.0313	0.093*	
H2N	0.4786	0.1644	0.8012	0.093*	
S11	0.58394 (9)	0.13158 (4)	1.3212 (4)	0.0673 (5)	

### Bis(pyridine-4-thioamide-κN)bis(thiocyanato-κN)zinc(II) (2) Atomic displacement parameters (Å^2^)

**Table:**

	*U*^11^	*U*^22^	*U*^33^	*U*^12^	*U*^13^	*U*^23^
Zn1	0.0576 (5)	0.0490 (4)	0.0632 (5)	−0.0008 (4)	0.000	0.000
N1	0.080 (4)	0.066 (3)	0.070 (4)	0.001 (3)	−0.001 (3)	−0.003 (3)
C1	0.063 (4)	0.060 (3)	0.057 (4)	0.003 (3)	−0.001 (3)	−0.003 (3)
S1	0.0802 (11)	0.0795 (10)	0.0724 (11)	0.0212 (9)	0.0063 (11)	−0.0055 (10)
N11	0.053 (3)	0.048 (2)	0.064 (3)	−0.0019 (19)	0.000 (3)	−0.006 (2)
C11	0.052 (3)	0.055 (3)	0.065 (4)	0.001 (2)	0.000 (3)	0.002 (3)
C12	0.053 (3)	0.056 (3)	0.071 (4)	0.003 (3)	0.000 (3)	0.007 (3)
C13	0.052 (3)	0.049 (2)	0.064 (3)	−0.002 (2)	0.001 (3)	−0.007 (3)
C14	0.058 (3)	0.052 (3)	0.075 (4)	0.003 (2)	0.010 (3)	0.000 (3)
C15	0.060 (3)	0.045 (2)	0.088 (5)	0.004 (2)	0.009 (4)	0.002 (3)
C16	0.054 (3)	0.054 (3)	0.061 (3)	0.001 (2)	0.004 (3)	−0.009 (3)
N12	0.052 (3)	0.066 (3)	0.067 (3)	−0.009 (2)	−0.001 (2)	−0.003 (3)
S11	0.0588 (9)	0.0727 (9)	0.0705 (10)	0.0005 (8)	0.0039 (8)	0.0091 (9)

### Bis(pyridine-4-thioamide-κN)bis(thiocyanato-κN)zinc(II) (2) Geometric parameters (Å, º)

**Table:**

Zn1—N1^i^	1.935 (6)	C12—H12	0.9500
Zn1—N1	1.935 (6)	C13—C14	1.376 (8)
Zn1—N11^i^	2.022 (5)	C13—C16	1.502 (8)
Zn1—N11	2.023 (5)	C14—C15	1.376 (9)
N1—C1	1.163 (8)	C14—H14	0.9500
C1—S1	1.617 (7)	C15—H15	0.9500
N11—C15	1.342 (8)	C16—N12	1.331 (8)
N11—C11	1.342 (7)	C16—S11	1.649 (7)
C11—C12	1.373 (8)	N12—H1N	1.0078
C11—H11	0.9500	N12—H2N	1.0347
C12—C13	1.389 (8)		
			
N1^i^—Zn1—N1	118.4 (4)	C13—C12—H12	120.2
N1^i^—Zn1—N11^i^	106.8 (2)	C14—C13—C12	117.8 (5)
N1—Zn1—N11^i^	105.9 (2)	C14—C13—C16	120.9 (5)
N1^i^—Zn1—N11	105.9 (2)	C12—C13—C16	121.3 (5)
N1—Zn1—N11	106.8 (2)	C13—C14—C15	119.8 (6)
N11^i^—Zn1—N11	113.3 (3)	C13—C14—H14	120.1
C1—N1—Zn1	176.5 (6)	C15—C14—H14	120.1
N1—C1—S1	177.8 (7)	N11—C15—C14	122.4 (6)
C15—N11—C11	117.9 (5)	N11—C15—H15	118.8
C15—N11—Zn1	119.9 (4)	C14—C15—H15	118.8
C11—N11—Zn1	122.1 (4)	N12—C16—C13	113.2 (6)
N11—C11—C12	122.5 (5)	N12—C16—S11	123.7 (5)
N11—C11—H11	118.8	C13—C16—S11	123.0 (5)
C12—C11—H11	118.8	C16—N12—H1N	125.8
C11—C12—C13	119.6 (5)	C16—N12—H2N	122.6
C11—C12—H12	120.2	H1N—N12—H2N	111.3

Symmetry code: (i) −*x*+3/2, −*y*+1/2, *z*.

### Bis(pyridine-4-thioamide-κN)bis(thiocyanato-κN)zinc(II) (2) Hydrogen-bond geometry (Å, º)

**Table:**

*D*—H···*A*	*D*—H	H···*A*	*D*···*A*	*D*—H···*A*
C15—H15···S1^ii^	0.95	2.96	3.690 (6)	135
N12—H1*N*···S11^iii^	1.01	2.41	3.358 (5)	156
N12—H2*N*···S1^iv^	1.03	2.41	3.424 (6)	166

Symmetry codes: (ii) −*x*+3/2, −*y*+1/2, *z*+1; (iii) *x*−1/4, −*y*+1/4, *z*−1/4; (iv) *x*−1/2, *y*, *z*+1/2.
