# Supplementary figures and images for: Crystal structures of tetra­kis­(pyridine-4-thio­amide-κN)bis­(thio­cyanato-κN)cobalt(II) monohydrate and bis­(pyridine-4-thio­amide-κN)bis­(thio­cyanato-κN)zinc(II)
Source: Acta Crystallogr E Crystallogr Commun. 2018 Jan 12;74(Pt 2):141–6. doi: 10.1107/S205698901800021X (PMC5956324; doi:10.1107/S205698901800021X)

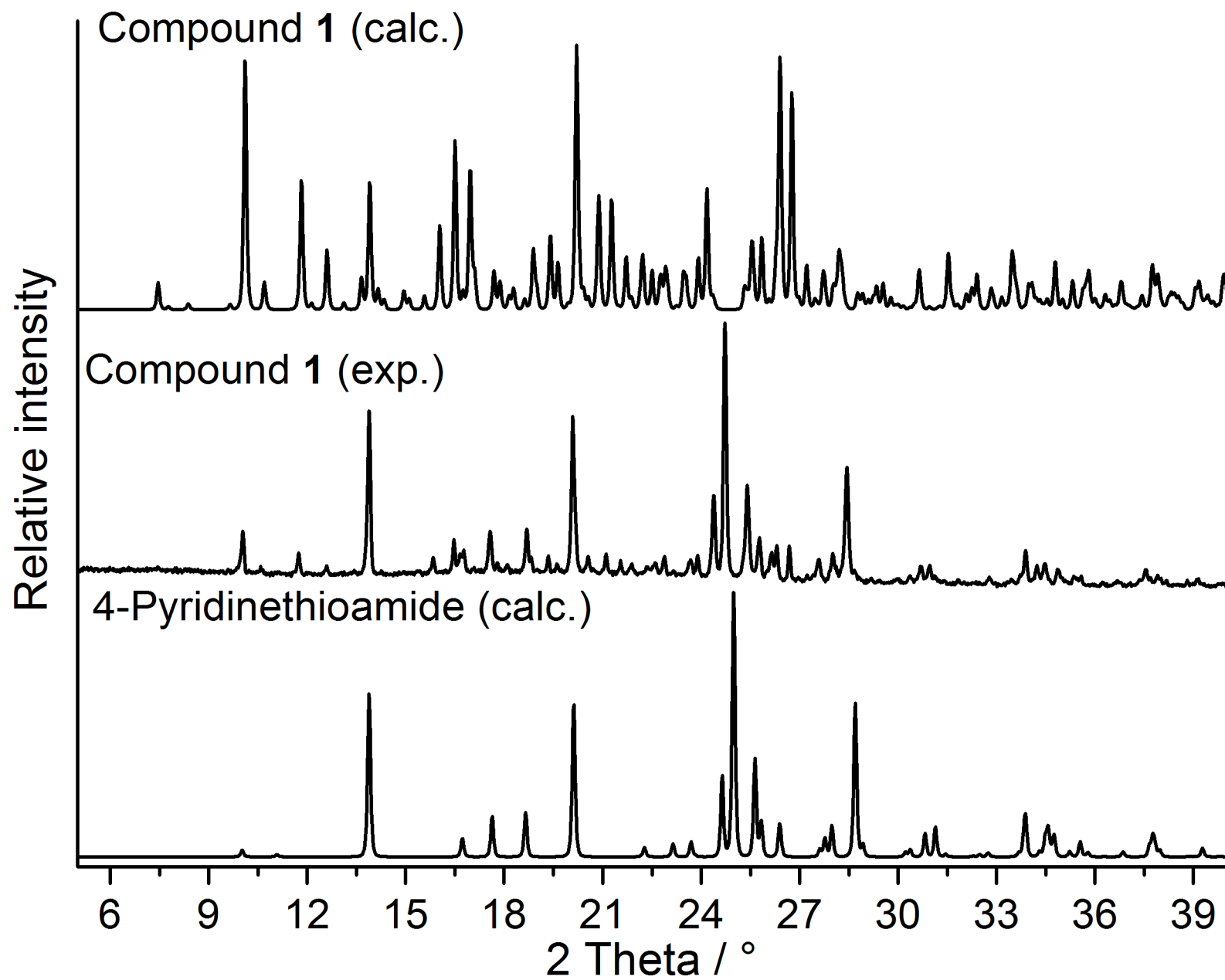

Supplement: Supplementary file 4 [file e-74-00141-sup4.pdf]

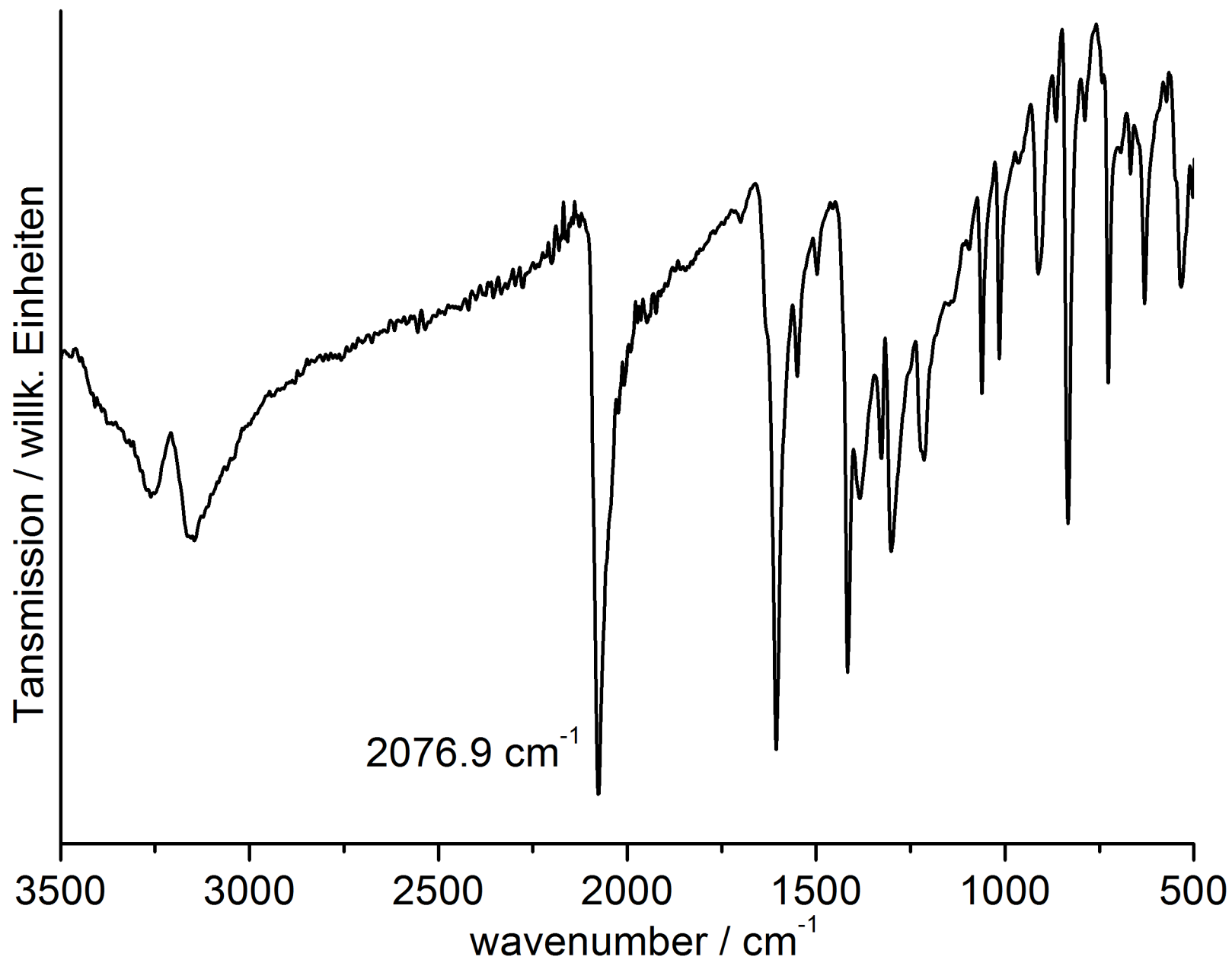

Supplement: Supplementary file 5 [file e-74-00141-sup5.pdf]
